# Optimized nursing strategies for skin graft outcomes: a narrative review of current evidence

**DOI:** 10.3389/fsurg.2026.1844508

**Published:** 2026-06-23

**Authors:** Wang Xing, Yi Huang, Jia Yao, Kan Jie, Tielong Xu

**Affiliations:** 1 Sichuan Province Forestry Center Hospital, Chengdu, China; 2 Evidence-Based Medicine Research Center Department, Jiangxi University of Chinese Medicine, Nanchang, China

**Keywords:** cell therapy, functional rehabilitation, negative pressure wound therapy, scar management, skin grafting, wound dressings

## Abstract

Skin grafting remains a cornerstone technique for repairing full-thickness skin defects. Its success depends on the coordinated optimization of graft survival, healing quality, functional recovery, and aesthetic outcomes. In recent years, a growing array of novel wound dressings, bioactive agents, cell-based therapies, and physical adjunctive modalities have expanded clinical options but simultaneously introduced complexity in balancing benefits against risks. In this review, we construct an evidence-based decision-making framework to guide clinical practice throughout the entire skin grafting process. We first focus on graft immobilization and adherence, critically appraising techniques to optimize graft survival. We then examine strategies for accelerated healing and pain reduction, comparing optimization approaches for donor and recipient sites. Finally, we analyze long-term efficacy and aesthetic outcomes, dissecting the trade-offs among different techniques in functional rehabilitation and scar management. By synthesizing high-quality evidence within this structured framework, this review aims to provide clinicians with a practical, evidence-anchored roadmap for individualized perioperative care.

## 1 Introduction

Skin grafting is a classic surgical method for repairing skin defects resulting from burns, trauma, ulcers, tumor resection, and other causes, and is widely utilized globally. With advancements in surgical techniques, skin grafting has evolved from a simple wound coverage procedure to a critical therapeutic approach that balances functional reconstruction with aesthetic restoration. However, the success of skin grafting is not a single surgical event but a continuous process encompassing graft survival, wound healing, infection control, pain management, functional rehabilitation, and scar prevention. The quality of postoperative nursing care directly impacts the ultimate surgical outcome and the patient's long-term quality of life.

Traditional skin graft nursing care often relies on conventional dressings and basic wound dressing changes, which have limitations in terms of healing speed, pain control, complication prevention, and long-term aesthetic results (1, 2). In recent years, with the cross-disciplinary advancement of materials science, regenerative medicine, bioengineering, and nursing science, perioperative management strategies for skin grafting have undergone significant transformation. Novel functional dressings (such as alginates, hydrocolloids, silver-containing dressings, and synthetic adhesive dressings) have emerged, demonstrating advantages in maintaining a moist environment, controlling exudate, reducing pain, and preventing infection. The evidence base for these dressings spans several decades, beginning with landmark randomized controlled trials that established the efficacy of synthetic adhesive dressings (2), hydrophilic polyurethane foam (1), and Biobrane® semi-synthetic dressings (3). More recent studies have further refined these findings, comparing different silver-containing formulations (4, 5), evaluating oxygen-diffusion dressings (6), and assessing patient-reported outcomes such as pain and comfort (7, 8). Together, this body of evidence—from early foundational RCTs to contemporary comparative trials—supports the role of functional dressings in modern skin graft care. Concurrently, bioactive agents [such as platelet-rich plasma, amniotic membrane, acellular dermal matrix (ADM), and fish skin grafts] and cell therapy techniques (such as ReCell®, micrografting) offer biological activity support and structural substitution for wound repair, not only accelerating healing but also making progress in reducing donor site morbidity and improving scar quality (9–20).

Furthermore, adjunctive therapies such as negative pressure wound therapy, hyperbaric oxygen therapy, energy-based therapies (shockwave, laser, ultrasound), and topical medications and plant extracts have further expanded the boundaries of skin graft nursing, shifting the care model from passive coverage to active modulation of the healing microenvironment (21–27). Perioperative systemic interventions—including preoperative tissue expansion, intraoperative hemostasis and harvesting technique optimization, and postoperative analgesia and anti-infection strategies—are also gradually forming standardized, individualized pathways, collectively providing crucial support for the success of modern skin grafting (28–35).

Despite the continuous emergence of new technologies, clinical practice still faces dilemmas in selection and controversies regarding efficacy. The effectiveness of different dressings, agents, and techniques varies significantly across wound types, patient populations, and healthcare settings. Their cost-effectiveness, ease of use, and long-term outcomes also require comprehensive evaluation. Therefore, conducting a narrative review and evaluation of the efficacy of various nursing techniques that promote skin graft success and rehabilitation, based on high-level clinical evidence, holds significant theoretical and practical importance. This review aims to synthesize the existed randomized controlled trials (RCTs), outlining the mechanisms of action, applicable scenarios, advantages, and limitations of various nursing techniques to provide an evidence-based foundation for clinical decision-making and promote the development of more precise, individualized, and comprehensive skin graft nursing care.

## 2 Literature search and selection

### 2.1 Literature search

To obtain high-quality evidence on nursing interventions for skin graft prognosis, the following electronic databases were searched: PubMed and Web of Science. The search period covered all articles published from database inception up to December 31, 2025.

The search strategy combined MeSH terms and free-text keywords as follows: For skin grafting: (“skin grafting” [MeSH] OR “skin graft* OR “skin transplantation” [MeSH]). For care-related interventions: (“nursing*” OR “care*” OR “management*” OR “rehabilitation*”). For the study type: randomized controlled trial.

Boolean operators (AND/OR) were used to combine the above terms. The detailed search logic was as follows: (“skin grafting” [MeSH] OR “skin graft*” OR “skin transplantation” [MeSH]) AND (“nursing*” OR “care*” OR “management*” OR “rehabilitation*”) AND “randomized controlled trial” [Publication Type]. Reference lists of retrieved articles were manually screened to identify additional relevant studies not captured by the electronic search.

### 2.2 Literature selection

The study selection process for identifying RCT evidence on skin graft nursing was as follows (36). After duplicate publications were deleted, two groups (group 1: Wang Xing and Jia Yao, group 2: Yi Huang and Kan Jie) independently performed the initial title and abstract screening. Full texts of potentially eligible studies and data extraction were then assessed independently by the same two groups. Disagreements were resolved by discussion or by consultation with the fifth author (Tielong Xu). The overall study selection and assessment process was supervised by Tielong Xu.

**Figure 1:**
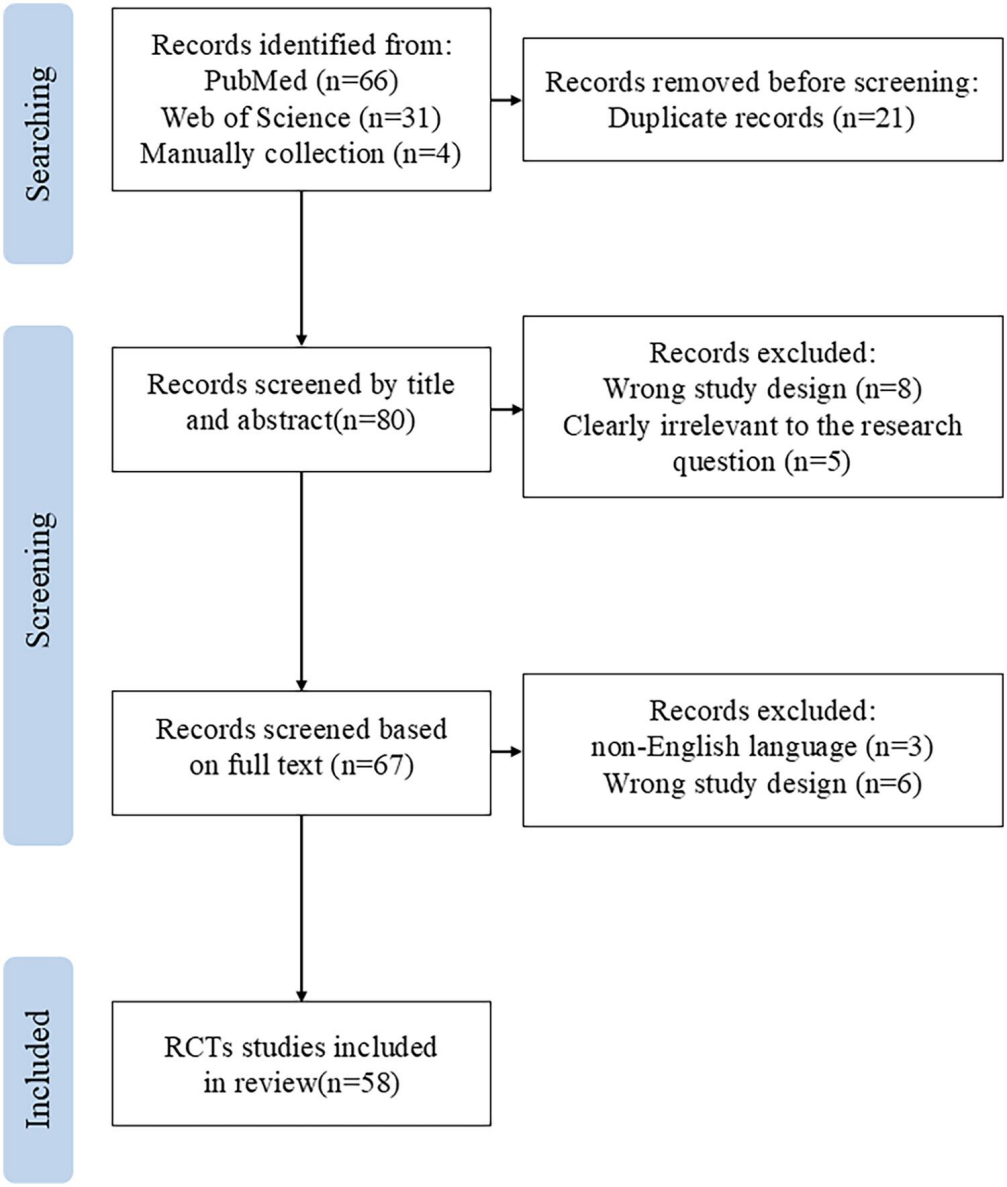
Flowchart of the study selection process.

A selection flow diagram summarizing the number of records identified, screened, excluded, and finally included is presented in Figure 1. The initial electronic search yielded 101 records. After removal of duplicates (*n* = 21), 80 records underwent title and abstract screening. Of these, 13 records were excluded as they were: Wrong study design (*n* = 8), clearly irrelevant to the research question (*n* = 5). The remaining 67 full-text articles were assessed for eligibility, of which 9 were excluded for reasons including: non-English language (*n* = 3), wrong study design (*n* = 6). Ultimately, 58 studies met the eligibility criteria and were included in this review.

## 3 Nursing techniques to enhance graft survival

Graft survival is the prerequisite for evaluating all outcomes following skin grafting. The core objectives at this stage are to ensure close adherence between the graft and the wound bed through reliable fixation techniques and to create ideal conditions for neo-vascularization by modulating the wound microenvironment.

### 3.1 Graft fixation

Traditional graft fixation commonly utilizes sutures or skin staples combined with external bolster dressings. Although widely used, this approach presents issues such as uneven pressure, inadequate drainage, and secondary trauma. Negative pressure wound therapy (NPWT), as a dynamic fixation method, demonstrates unique value, but its efficacy is highly context-dependent. A prospective randomized controlled trial in burn patients undergoing split-thickness skin grafting (STSG) showed that NPWT dressings resulted in significantly higher graft take rates compared to the conventional Vaseline gauze group (mean 96.7% vs. 87.5%) and reduced dressing change time (21). However, in routine skin graft sites where baseline wound management is already adequate, the benefit of NPWT on graft take may be limited, with its advantages more evident in pain reduction and improved patient satisfaction (22).

In the field of biological fixation, the amniotic membrane offers distinct advantages as a natural biological membrane. Clinical trials have demonstrated that fixation using amniotic membrane wrapping significantly improves the complete graft take rate in limb burns, shortens the time to graft take, and avoids the secondary trauma associated with staple removal compared to skin staple fixation, making it particularly suitable for pediatric patients (9). This effect is attributed not only to physical fixation but also to the favorable bioactive microenvironment provided by the amniotic membrane.

When applying NPWT to skin graft recipient sites, the optimal negative pressure setting is typically −75 to −125 mmHg delivered continuously or intermittently (21). For STSG, continuous negative pressure at −75 to −80 mmHg is commonly used to avoid shear stress and graft displacement, whereas higher pressures (up to −125 mmHg) may be applied for thicker grafts or wound beds with moderate exudate (22). In the studies cited (21, 22), continuous NPWT at −80 mmHg was used and was well tolerated without graft shearing or excessive pain. Intermittent pressure cycling (e.g., 5 min on/2 min off) may be considered for wounds requiring additional granulation tissue stimulation, though continuous mode is preferred immediately post-grafting to maximize adherence.

### 3.2 Nursing techniques for anti-infection and angiogenesis promotion

Infection is the leading cause of skin graft failure. Silver-containing dressings are a crucial choice for wounds at high risk of infection due to their broad-spectrum antimicrobial activity. Studies have shown that Acticoat™ silver-coated dressings provide comparable infection control to 1% silver sulfadiazine in partial-thickness burns (4). Additionally, Biobrane® does not increase infection risk when used for partial-thickness burns, suggesting its reliability in providing antimicrobial protection (3). A thermoreversible wound covering gel (Traumasert®) has been shown to be superior to traditional silver sulfadiazine cream in wound assessment and leakage control (37), indirectly indicating its potential value in infection prevention and control.

However, the selection of silver-containing dressings requires caution. A prospective comparison revealed that the healing time for Biatain Alginate Ag was significantly shorter than for Biatain Ag (5), indicating that even among silver-impregnated dressings, the carrier material significantly influences the healing process. For wounds requiring full-thickness coverage, skin graft substitutes like hyaluronic acid matrices, while avoiding donor site morbidity, necessitate a careful trade-off considering potential increased infection risk and home care burden (38).

For wounds with a compromised bed, hyperbaric oxygen therapy can play a crucial adjunctive role by increasing tissue oxygen tension, enhancing leukocyte bactericidal capacity, and promoting angiogenesis. A randomized controlled trial involving patients undergoing medium-thickness skin grafting for post-traumatic wounds demonstrated that postoperative adjunctive hyperbaric oxygen significantly improved early graft survival (23). For complex wounds, retrospective studies indicate that NPWT can significantly promote granulation tissue formation in replanted limb wounds, shorten the waiting time for secondary surgery, and reduce the need for flap transplantation (39), indirectly creating favorable conditions for subsequent graft survival. Furthermore, for skin graft sites with extremely high infection risk, such as the nose, short-term oral azithromycin has been shown to significantly improve early graft survival (28). Concurrently, smoking has a significant negative impact on graft survival, highlighting the necessity of patient behavioral intervention (28) (Table 1).

**Table 1:** Optimization strategies for graft survival.

Optimization strategy	Used methods	Key evidence	Clinical considerations
Dynamic fixation	Negative pressure wound therapy (NPWT)	Higher graft survival in burn patients vs. Vaseline gauze (21). In routine wounds, benefits more in pain reduction than graft take (22).	Highly effective for compromised wounds. Limited added benefit if baseline care is adequate.
Biological fixation	Amniotic membrane wrapping	Improves graft take in limb burns, shortens time to take, avoids staple trauma. Ideal for children (9).	Provides fixation and bioactive environment. Good for vulnerable patients.
Anti-infection	Silver-containing dressings	Infection control similar to 1% silver sulfadiazine in burns (4). Carrier type (e.g., alginate vs. foam) affects healing time (5).	Choose dressing based on antimicrobial need and carrier effect on healing.
	Short-term oral antibiotics (e.g., azithromycin)	Improves early graft survival in high-risk sites like the nose (28).	Targeted use for high-risk anatomical locations.
Anti-infection & angiogenesis	Hyperbaric oxygen therapy	Improves early graft survival in post-traumatic wounds (23).	Adjunctive use for wounds with poor beds. Boosts leukocyte function and blood vessel growth.
Wound bed preparation	NPWT	Promotes granulation tissue, shortens time to surgery, reduces need for flaps in complex wounds (39).	Prepares wound bed, indirectly improving graft survival.
Patient behavioral intervention	Smoking cessation	Smoking negatively impacts graft survival (28).	Essential component of perioperative care.

### 3.3 Graft-type specific considerations for survival

Skin graft survival is influenced not only by postoperative nursing techniques but also by the intrinsic biological and mechanical properties of the graft itself. The three most used graft types in clinical practice are STSG, full-thickness skin grafts (FTSGs), and composite grafts. Each has distinct survival characteristics, healing kinetics, and nursing implications.

STSGs are the most used graft type. Their thinness (0.008–0.015 inches) allows for rapid revascularization and makes them forgiving of minor nursing imperfections; however, they are more prone to contraction and have poorer aesthetic outcomes. The evidence synthesized in this review predominantly derives from STSG studies, demonstrating that NPWT improves take rates in burns (21) and that silver-containing dressings effectively control infection without delaying healing (4, 5). For donor site management, moist dressings and cell suspension technologies (e.g., ReCell®) have significantly reduced donor site morbidity (10–12, 40, 41).

FTSGs are indicated for cosmetically sensitive areas such as the face and nose. Unlike STSG, FTSG lacks a dermal plexus and relies entirely on recipient bed neovascularization, making them more susceptible to ischemic necrosis. A randomized controlled trial on full-thickness nasal grafts demonstrated that short-term oral azithromycin significantly improved graft survival, highlighting the critical importance of infection control for this graft type (28). The same study confirmed that smoking has a pronounced negative impact on FTSG survival (28). Nursing priorities for FTSG should therefore emphasize strict immobilization, antimicrobial stewardship, and smoking cessation counseling.

Composite grafts, which include skin plus underlying cartilage or fat, are occasionally used for complex reconstructions such as nasal alar or ear defects. They rely on both plasmatic imbibition and direct vascular anastomosis from the recipient bed, making them the most challenging in terms of survival. Nursing strategies for composite grafts should emphasize meticulous hemostasis, strict immobilization, and avoidance of any pressure or shear forces. Although high-level RCTs on composite graft nursing are limited, principles derived from FTSG and flap care—such as minimizing edema and optimizing perfusion—are commonly applied.

In summary, the choice of graft type fundamentally affects the goals and intensity of postoperative nursing care. STSGs benefit most from interventions that control exudate and prevent infection (e.g., NPWT, silver dressings). FTSGs require enhanced attention to vascular support, infection prophylaxis, and patient risk modification (e.g., smoking cessation). Composite grafts demand the most stringent immobilization and perfusion monitoring. Future RCTs should directly compare nursing protocols across graft types to establish type-specific evidence-based guidelines.

## 4 Nursing techniques to accelerate healing and reduce pain

Once graft survival is achieved, clinical focus shifts to accelerating wound healing and enhancing patient comfort during the treatment process. This phase necessitates considering the donor and recipient sites holistically, as donor site morbidity itself is a major source of patient discomfort.

### 4.1 Strategies for optimizing donor site healing

The donor site, as a “necessary injury,” directly impacts the overall patient recovery process when it heals. Novel dressings based on the principle of moist wound healing have become the foundation of donor site care. A randomized controlled trial demonstrated that the time to re-epithelialization was significantly shorter with a bovine collagen-calcium alginate dressing combined with a transparent film compared to traditional saline-soaked gauze (40). In pediatric patients, calcium alginate dressings outperformed hydrofiber and foam dressings in terms of healing time (41). Hydrophilic polyurethane foam dressings showed no statistically significant difference in complete epithelialization rate compared to Vaseline gauze (1). Telfa AMD® demonstrated superior re-epithelialization time compared to traditional chlorhexidine-impregnated Vaseline gauze (8). In the treatment of partial-thickness burns, the semi-synthetic dressing Biobrane® achieved a significantly shorter mean healing time than 1% silver sulfadiazine without increasing infection risk (3). Furthermore, Moist Exposed Burn Ointment (MEBO) was significantly superior to semi-permeable film dressings in promoting anatomical healing and accelerating the restoration of physiological barrier function in partial-thickness donor sites (42).

Cell therapy and micrografting techniques represent significant advancements in donor site management. Technologies like ReCell®, which involve preparing a cell suspension from a small donor skin sample to cover a larger recipient area, have shown comparable healing rates to traditional STSG in treating deep partial-thickness burns (10, 11). A recent multicenter randomized controlled trial further confirmed that using this technique combined with widely meshed grafts achieved comparable healing rates to traditional skin grafting while reducing the donor skin harvesting area by an average of 27.4% (12). This “minimally invasive” characteristic directly translates to reduced donor site pain and accelerated healing. Similarly, re-using discarded intraoperative skin grafts as finely divided skin grafts on the donor site has also been shown to effectively accelerate donor site healing (43). However, the application of cell therapy in specific chronic wounds requires careful evaluation. An open-label randomized controlled trial involving well-vascularized diabetic foot wounds larger than 6 cm^2^ showed that ReCell® “spray-on” autologous skin transplantation did not demonstrate significant superiority over standard care in terms of complete healing rates at 6 and 12 months, with similar healing trajectories observed (44). Stamp skin grafting, a classic micrografting technique, has shown clear benefits in outpatient care for chronic wounds (such as venous leg ulcers, postoperative skin defects, and diabetic foot ulcers), significantly shortening healing time and improving healing efficacy (45). Epidermal grafting, such as ultra-thin skin grafting, also features minimally invasive characteristics, reducing the impact on donor site function (46) and contributing to reduced patient pain compared to conventional dressings due to its minimally invasive nature (46).

Moreover, various physical therapies have demonstrated significant advantages in promoting donor site healing. A prospective trial confirmed that a single session of low-energy extracorporeal shock wave therapy applied immediately after donor site harvesting significantly shortened the mean time to complete epithelialization (24). Low-level laser therapy accelerated early healing of skin graft donor sites in burn patients (25). Non-contact low-frequency ultrasound combined with standard care significantly shortened the mean healing time and increased the proportion of complete epithelialization at donor sites (26). Continuous direct current anodal microcurrent therapy shortened the mean time to 95% wound closure from 7.2 to 4.6 days (27).

In the field of chronic, hard-to-heal wounds, acellular fish skin grafts have accumulated robust evidence. Multiple high-quality randomized controlled trials consistently demonstrate that fish skin grafts significantly improve complete healing rates, accelerate wound area reduction, and achieve shorter mean healing times compared to standard care (such as collagen-alginate dressings) (13–16). Although the initial cost per treatment may be higher, the overall annualized treatment cost is more favorable due to effective healing promotion and reduced long-term care burden and complication risks, demonstrating good cost-effectiveness (14).

Topical medications and plant extracts also exert direct effects in promoting healing. Studies have confirmed that topical application of the anti-fibrotic drug pirfenidone gel significantly improves the epithelialization rate and increases neo-epidermal thickness in the early postoperative period at donor sites (47). Natural plant extracts also show application potential: birch bark extract gel was shown to safely and effectively shorten donor site wound closure time in a Phase III clinical trial (48); an ointment containing active ingredients like Alkannin also significantly accelerated donor site healing, achieving a very high complete healing rate (49) (Table 2).

**Table 2:** Optimization strategies for donor site healing.

Optimization strategy	Used methods	Key evidence	Clinical considerations
Moist wound healing	Novel dressings (e.g., collagen-alginate, calcium alginate, hydrofiber, polyurethane foam)	Faster healing than saline gauze or vaseline (1, 40, 41). Biobrane® heals faster than silver sulfadiazine without raising infection risk (3).	Foundation of donor site care. Choice depends on factors like exudate level, cost, and patient comfort.
Cell therapy & micrografting	ReCell® (autologous skin cell suspension)	Healing similar to traditional STSG in deep burns; reduces donor area by ∼27% (10–12).	Less invasive, less donor site pain. Limited efficacy in some chronic wounds (44).
	Stamp skin grafting	Faster healing in chronic wounds like venous leg ulcers and diabetic foot ulcers (45).	Effective and practical for chronic wound management in outpatient care.
Physical therapy	Extracorporeal shock wave therapy (single session)	Shortens time to full healing when applied right after harvest (24).	Simple, one-time intervention with significant benefits.
	Low-level laser therapy	Speeds early healing of donor sites in burn patients (25).	Non-invasive adjunct to standard care.
	Non-contact low-frequency ultrasound	Shortens healing time and increases complete healing when combined with standard care (26).	Enhances healing in difficult-to-heal donor sites.
	Continuous direct current anodal microcurrent	Cuts time to 95% closure from 7.2 to 4.6 days (27).	Rapidly accelerates wound closure.
Bioactive materials	Acellular fish skin graft	Improves healing rates, speeds wound reduction, and shortens healing time in chronic wounds vs. standard care (13–16).	Cost-effective long-term despite higher upfront cost.
	Nanofibrillar cellulose (NFC) dressing	Faster re-epithelialization vs. Suprathel® in burn donor sites; self-detachment (50)	Biocompatible, xeno-free; ideal for early detachment; no intrinsic antimicrobial effect.
Topical medications	Pirfenidone gel	Improves epithelialization rate and increases neo-epidermal thickness at donor sites (47).	Direct pharmacological promotion of healing.
Plant extracts	Birch bark extract gel	Safely shortens donor site closure time (48).	Natural extract with proven efficacy in Phase III trial.
	Alkannin-containing ointment	Speeds donor site healing with high complete healing rate (49).	Traditional remedy with clinical benefit.

In addition to the foregoing dressings, nanofibrillar cellulose (NFC), a wood-based, xeno-free biomaterial, has recently been evaluated for skin graft donor site treatment in burn patients. In a clinical trial involving nine burn patients, an NFC wound dressing was compared with a lactocapromer-based dressing (Suprathel®). The NFC dressing self-detached from the epithelialized donor site on average at postoperative day 18, compared with day 22 for Suprathel®, indicating faster re-epithelialization. Moreover, NFC dressing demonstrated good biocompatibility, appropriate adherence, and no allergic reactions. Its high hydrophilicity and ability to form a water film between the dressing and wound bed may create a favorable environment for skin regeneration while preventing tissue integration. Although NFC does not possess intrinsic antimicrobial activity, it does not support bacterial growth and can potentially serve as a carrier for bioactive agents or cells. These preliminary results suggest that NFC dressing is a promising option for donor site management, particularly when early detachment and patient comfort are prioritized (50).

### 4.2 Recipient site healing and pain management

At the recipient site, dressing choice similarly impacts healing speed and patient experience. Synthetic adhesive dressings generally provide better pain control than traditional gauze. Early studies indicated that patients treated with water vapor-permeable synthetic dressings (e.g., Tegaderm, Op-Site) had significantly lower mean pain scores compared to those treated with fine-mesh gauze (2). Subsequent studies further confirmed that adhesive dressings (Mefix®) resulted in lower early postoperative pain scores compared to calcium alginate dressings (7); a polyethylene non-adherent film was also superior to chlorhexidine-impregnated Vaseline gauze in terms of postoperative comfort and the proportion of patients experiencing low pain levels (51). Medicated dressings like Biatain-Ibu demonstrated targeted analgesic advantages, with superior pain control compared to other synthetic dressings (52). A novel oxygen-diffusion dressing significantly reduced pain scores at multiple postoperative time points compared to Vaseline gauze (6); hydrophilic polyurethane foam dressings significantly lowered pain intensity in the early postoperative period, also outperforming Vaseline gauze (1). Telfa AMD® yielded better pain scores than traditional chlorhexidine-impregnated Vaseline gauze (8).

Negative pressure dressings also play an important role in pain management at the recipient site. A randomized controlled trial showed that NPWT significantly reduced pain compared to conventional wet-to-dry dressings and received higher ratings for dressing conformity, ease of use, and overall satisfaction (22). However, the impact of NPWT on healing time at the recipient site varies. In acute burn care, NPWT did not shorten overall healing time but significantly reduced the number of dressing changes, offering care convenience (53). When selecting a negative pressure wound therapy system, cost-effectiveness analysis should inform the decision if efficacy is comparable (54). In the outpatient treatment of partial-thickness burns, the semi-synthetic dressing Biobrane® significantly reduced pain scores and analgesic use compared to 1% silver sulfadiazine ointment (3) (Table 3).

**Table 3:** Optimization strategies for recipient site healing and pain management.

Optimization strategy	Used methods	Key evidence	Clinical considerations
Pain control & healing	Synthetic adhesive dressings (e.g., Tegaderm, Op-Site, Mefix®)	Lower pain scores vs. fine-mesh gauze or calcium alginate (2, 7).	Superior comfort and pain control for recipient sites.
	Novel oxygen-diffusion dressing	Lower pain scores post-op vs. Vaseline gauze (6).	Supports healing while reducing pain.
	Hydrophilic polyurethane foam dressings	Significantly lowered pain intensity in the early postoperative period, outperforming Vaseline gauze (1).	Good option for managing exudate while providing pain relief.
Pain control	NPWT	Less pain than wet-to-dry dressings; better comfort and satisfaction (22).	Beneficial for pain management, though its effect on healing time may vary; can reduce number of dressing changes (53).
	Biobrane® (semi-synthetic dressing)	Less pain and analgesic use than silver sulfadiazine in outpatient burns (3).	Ideal for outpatient management of burns.
	Medicated dressings (e.g., Biatain-Ibu)	Better pain control than other synthetic dressings (52).	Combines wound care with local pain management.
Anti-infection & healing	Silver sulfadiazine + hyaluronic acid (SSD-HA)	Effective in acute wounds, but lower complete healing rate vs. polyhexanide-plant extract (55)	Useful for infected recipient sites; alternative dressings may be preferred when faster healing or fibrin reduction is needed.

Silver sulfadiazine (SSD) has long been used for its broad-spectrum antimicrobial activity, but concerns regarding delayed wound healing have prompted combination with other bioactive molecules. Hyaluronic acid (HA), a key extracellular matrix component, has been combined with SSD to create an advanced dressing (Connettivina® Bio Plus). A randomized trial comparing this HA-SSD combination with a polyhexanide-Triticum vulgare extract (Fitostimoline® Plus) in acute superficial skin wounds found that while both dressings were effective and well tolerated, the polyhexanide-plant extract combination achieved a significantly higher rate of complete wound healing (93.3% vs. 56.6%, *p* = 0.001) and was more effective in reducing fibrin in the wound bed (*p* = 0.047). Although this study did not specifically enroll skin graft donor or recipient sites, the findings are relevant because acute wounds share similar healing requirements. The HA-SSD combination remains a valid option for infected or high-risk wounds, but clinicians should be aware that alternative formulations may offer superior healing outcomes, particularly when fibrin accumulation is present (55).

## 5 Techniques for optimizing long-term outcomes and aesthetic results

The ultimate success of skin grafting hinges not only on early survival and healing but also on long-term functional recovery and scar aesthetics. Intervention strategies at this stage require careful trade-offs between short-term efficacy and long-term outcomes.

### 5.1 Rehabilitation of functionally critical areas

For areas involving joints or the hand, promoting functional recovery is the core objective. Negative pressure dressings have shown unique value in this domain. Multiple prospective randomized controlled trials focusing on the radial forearm free flap donor site (a functionally critical area) consistently demonstrated that NPWT significantly accelerated postoperative recovery of grip strength (56) and improved early hand function and wrist range of motion (57). Although NPWT did not show significant advantages in reducing graft-related complications, decreasing wound area, or improving scar aesthetics (56–58), its clear benefits in functional rehabilitation make it a preferred management option for postoperative care in such areas.

Tissue expansion techniques eliminate donor site functional morbidity by avoiding the need for skin grafting altogether. A cohort study focusing on the radial forearm donor site showed that using a non-invasive tissue expansion device like DynaClose significantly reduced the need for STSG (by 93%) and substantially lowered total wound care costs (29). For specific localized full-thickness acute burn wounds, intraoperative application of skin stretching devices for primary closure can serve as an alternative to STSG (30), also avoiding donor site morbidity and its impact on functional recovery.

Innovations in skin harvesting techniques also influence functional rehabilitation. Medium-thickness skin grafts harvested using a molecular resonance generator exhibited faster donor site healing, fewer follow-up visits, and lower complication rates (31), aiding in early donor site functional recovery. The synthetic dressing Spincare® (a portable electrospun nanofiber polymer matrix) allows for early showering, helping patients resume activities of daily living sooner (32). Traumasert® (a thermoreversible wound covering gel) facilitates the filling of irregular wounds and is superior to traditional dressings in wound assessment and leakage control (37), offering improved nursing options for patients with wounds located in functional areas (Table 4).

**Table 4:** Optimization strategies for rehabilitation of functionally critical areas.

Optimization strategy	Used methods	Key evidence	Clinical considerations
Functional rehabilitation	NPWT	Faster grip strength recovery (56) and improved early hand function after radial forearm flap harvest (57).	Good for donor sites in functional areas like hand/wrist.
Donor site morbidity avoidance	Tissue expansion (e.g., DynaClose)	Reduces need for STSG by 93% and lowers costs for radial forearm donor sites (29).	Avoids grafting and functional loss.
	Intraoperative skin stretching devices	Alternative to STSG for small, full-thickness acute burn wounds (30).	Avoids donor site morbidity for small wounds.
Early functional recovery	Molecular resonance generator (for skin harvesting)	Medium-thickness grafts show faster healing, fewer visits, and lower complications (31).	Harvesting technique aids faster recovery.
	Spincare® (Electrospun Nanofiber Matrix)	Allows early showering, helping patients resume daily activities sooner (32).	Facilitates early return to normal function through enhanced dressing convenience.

### 5.2 Scar prevention and management

Scar quality is a core indicator for evaluating long-term aesthetic outcomes. Various techniques show potential in improving scar quality, but this often involves trade-offs with short-term efficacy or potential risks.

#### 5.2.1 Scar improvement with biological agents

Allogeneic skin has demonstrated clear value in improving scar quality. A study on deep second-degree facial burns showed that early application of glycerol-preserved allograft resulted in no significant hypertrophic scarring at 3 and 6 months postoperatively, whereas scarring occurred in the standard treatment group (17). The effect of platelet-rich plasma on scar quality appears variable, showing improvement in donor site scars (18) but no significant effect in deep dermal burn recipient sites (19).

#### 5.2.2 Structural substitution

When ADMs (e.g., Novomaix) are used in combination with STSG, although early healing may be slower compared to STSG alone, they can significantly improve the elasticity and extensibility of long-term scars—objective parameters that are associated with better functional and aesthetic outcomes (softer, more pliable scars). However, current evidence does not demonstrate a statistically significant superiority of ADM over STSG alone in terms of pigmentation, vascularity, or overall cosmetic scar scores (20). This highlights the need for clinicians to communicate clearly with patients, making a choice between “healing speed” and “functional scar quality.” For wounds requiring full-thickness coverage, skin graft substitutes like hyaluronic acid matrices achieve comparable long-term scar aesthetics to full-thickness skin grafts (38), providing an option to avoid donor site morbidity without sacrificing long-term aesthetics.

Beyond improving scar quality, ADMs have also expanded the clinical indications for skin grafting to include wounds traditionally considered unsuitable for STSG, such as those failed in the standard of care or with exposed bone, tendon, or joint capsule (59, 60). By providing a temporary vascularized scaffold that promotes granulation tissue formation and neovascularization, ADMs create a receptive wound bed that can support subsequent skin graft take. This two-stage approach (ADM placement followed by delayed STSG) has enabled limb salvage and functional reconstruction in complex injuries where flap surgery might have been the only alternative (59). Clinicians should be aware that while ADM expands treatable indications, it requires careful patient selection, longer treatment duration, and higher initial costs—trade-offs that must be discussed with the patient.

#### 5.2.3 Silver-containing dressings

A prospective comparative study revealed significant differences in long-term outcomes between different silver-containing dressings. Although Biatain Ag (silver foam) provided better early postoperative pain control than Biatain Alginate Ag (silver alginate), its scar quality scores were significantly worse at 6 months post-surgery (5). This finding has significant clinical implications: if a patient has high aesthetic demands for the donor site or is at high risk for post-surgical scarring, silver alginate may be the preferred choice; however, if a patient has heavy exudate and high pain sensitivity, silver foam may be more advantageous for early management.

#### 5.2.4 Donor site scar management

Re-using discarded intraoperative skin grafts as finely divided skin grafts on the donor site has been shown to not only accelerate healing but also improve scar appearance (43), representing a cost-effective strategy for donor site scar management. Semi-permeable film dressings were significantly inferior to Moist Exposed Burn Ointment in promoting anatomical healing and accelerating the restoration of physiological barrier function at partial-thickness donor sites, with the latter resulting in superior long-term scar quality (42).

#### 5.2.5 Topical medications and plant extracts

These agents show potential in scar prevention and management. Birch bark extract gel, in a Phase III clinical trial, not only safely and effectively shortened donor site wound closure time but also improved long-term skin appearance (48). This finding supports the application of natural extracts in scar management.

#### 5.2.6 Cost vs. effectiveness of advanced dressings

Several comparative studies have indicated that different advanced dressings, including Mefix®, Biatain-Ibu, Dressilk, Biobrane®, and Suprathel®, may show no statistically significant differences in scar quality (7, 52, 61). Suprathel was optimal in terms of nursing workload but had the highest cost (7, 52, 61). Compared to traditional Vaseline gauze, a novel oxygen-diffusion dressing also showed no significant difference in long-term cosmetic outcomes (6). These findings suggest that in the absence of clear long-term benefits, cost-effectiveness analysis should be a consideration in clinical decision-making (Table 5).

**Table 5:** Optimization strategies for scar prevention and management.

Optimization strategy	Used methods	Key evidence	Clinical considerations
Scar improvement	Allogeneic skin	Early use on deep facial burns led to no significant scarring at 3–6 months, unlike standard care (17).	Clear value in improving long-term scar quality, especially in high-visibility areas.
	ADM (e.g., Novomaix)	With STSG, improves scar elasticity and extensibility, though early healing may be slower (20).	Requires trade-off: “healing speed” vs. “functional scar quality.”
	Moist exposed burn ointment (MEBO)	Better anatomical healing and scar quality vs. semi-permeable film dressings (42).	Dressing choice influences both healing and scar outcome.
	Birch bark extract gel	Shortened closure time and improved long-term skin appearance in Phase III trial (48).	Natural extract with dual benefits for healing and aesthetics.
Scar management trade-off	Silver-containing dressings	Silver alginate gave better scar scores at 6 months vs. silver foam, which offered better early pain control (5).	Choice should be based on patient priorities: long-term aesthetics vs. short-term pain/exudate management.
Donor site scar management	Re-used minced skin grafts	Accelerates healing and improves scar appearance of the donor site (43).	Cost-effective strategy that turns waste tissue into a scar-management tool.
Cost-effectiveness	Advanced dressings (e.g., Mefix®, Biatain-Ibu, Dressilk, Biobrane®, Suprathel®)	No major differences in scar quality among several advanced dressings (7, 52, 61). Suprathel had lowest nursing workload but highest cost.	Use cost-effectiveness when long-term scar benefits are similar.

## 6 Comprehensive decision flowchart for postoperative skin graft nursing

**Figure 2:**
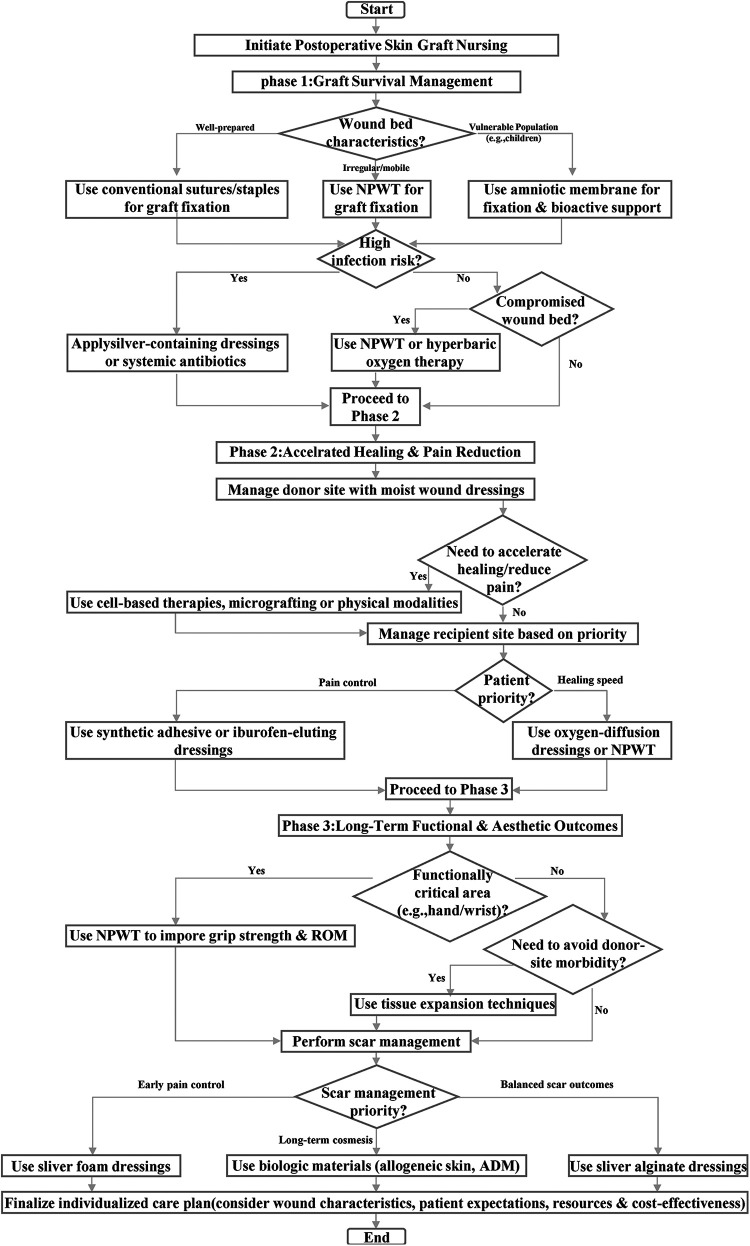
A comprehensive decision flowchart based on the evidence synthesized in this review.

Based on the evidence synthesized in this review, a comprehensive decision flowchart (Figure 2) is proposed to integrate the key strategies across the three critical phases of skin graft postoperative care: graft survival, healing and pain control, and long-term functional and aesthetic outcomes. The flowchart is designed to assist clinicians in navigating the growing array of interventions by structuring decisions according to phase-specific goals and patient-wound characteristics.

### 6.1 Phase 1: graft survival

The primary objective is to secure graft adherence and promote neovascularization. For graft fixation, conventional sutures or staples suffice for well-prepared wound beds, whereas NPWT is preferred for irregular or mobile areas and has been shown to improve graft take rates (21). In vulnerable populations (e.g., children) or to avoid secondary trauma, amniotic membrane offers both mechanical fixation and a bioactive microenvironment (9). Infection prevention and angiogenesis support are addressed by selecting silver-containing dressings or systemic antibiotics when infection risk is high (4, 5, 28), and by employing NPWT or hyperbaric oxygen therapy when the wound bed is compromised (23, 39). In the graft survival phase, when NPWT is selected for recipient site fixation, we recommend an initial continuous negative pressure of −75 to −80 mmHg for the first 48–72 h post-grafting, followed by reassessment. Pressure should be reduced if the patient experiences significant pain or if graft blanching indicates excessive compression (21, 22).

### 6.2 Phase 2: accelerated healing and pain reduction

This phase requires simultaneous management of donor and recipient sites. For donor sites, moist wound healing dressings form the foundation (1, 40, 41), while cell-based therapies (e.g., ReCell®), micrografting, and physical modalities (extracorporeal shock wave, low-level laser, ultrasound) can further accelerate re-epithelialization and reduce pain (10–12, 24–27, 43, 45). At the recipient site, dressings are chosen based on patient priorities: synthetic adhesive or ibuprofen-eluting dressings prioritize pain control (2, 7, 52), whereas oxygen-diffusion dressings or NPWT may be favored when optimizing healing speed is paramount (1, 6, 22).

### 6.3 Phase 3: long-term functional and aesthetic outcome

In functionally critical areas such as the hand or wrist, NPWT has demonstrated benefits in accelerating recovery of grip strength and range of motion (56, 57); tissue expansion techniques can obviate the need for skin grafting altogether, thereby eliminating donor-site morbidity (29, 30). Scar management involves a deliberate trade-off between short-term efficacy and long-term cosmesis. Biologic materials (allogeneic skin, ADMs) generally improve scar quality, albeit sometimes at the cost of slower early healing (17, 20). Even among silver dressings, silver alginate yields superior scar outcomes compared to silver foam, which provides better early pain control—an example of the trade-off that should be discussed with the patient (5).

### 6.4 Integrated decision-making

All interventions ultimately converge on individualized choices that consider wound characteristics, patient expectations, healthcare resources, and cost-effectiveness (7, 14, 52, 54, 61). The flowchart organizes these three phases and their respective decision nodes into a coherent visual framework, enabling clinicians to quickly identify the appropriate strategies for each stage while maintaining a holistic view of the patient's journey. It emphasizes that optimal skin graft care is not a linear protocol but a dynamic process of balancing multiple goals across the perioperative continuum.

## 7 Discussion and future directions

Current evidence indicates that the success of skin grafting and improvement in patient rehabilitation outcomes increasingly rely on personalized, multimodal comprehensive care strategies. A comprehensive evaluation of existing research can provide clear guidance for clinical decision-making and illuminate future development directions. Optimizing skin graft prognosis is a systematic endeavor requiring a continuous process spanning pre-, intra-, and postoperative phases. Based on existing evidence, the following clinical decision-making framework can be outlined.

### 7.1 Selecting dressings based on multidimensional indicators

Novel functional dressings have established their role in improving patient experience and optimizing the healing process. Selection should go beyond the single indicator of “healing time” to incorporate a multidimensional trade-off: patient comfort and pain management are core considerations; ease of use and cost-effectiveness are equally important; wound characteristics and assessment needs dictate the dressing type. Results from large-scale multicenter direct comparison trials, such as the Rembrandt trial, will provide crucial evidence for ranking dressing efficacy and informing clinical recommendations (62). Clinical decisions should be refined based on wound type, individual patient needs, and available healthcare resources. Specifically, for highly exuding burns with a compromised wound bed, NPWT is the preferred method to enhance graft take; for routine wounds, NPWT may be more valuable for pain reduction and early functional improvement. For complex wounds, NPWT effectively promotes granulation tissue formation, creating conditions for subsequent grafting (39).

### 7.2 Biologics and advanced therapies: potential to shift from adjunctive to core options

Bioactive agents and cell therapies are evolving from adjunctive roles towards core treatment options. For hard-to-heal wounds, acellular fish skin grafts show significant advantages in healing rates and long-term cost-effectiveness; in acute burns, allogeneic skin accelerates re-epithelialization and improves scar quality; in reducing donor site morbidity, technologies like ReCell® and ultra-thin grafting represent a direction towards minimally invasive approaches. However, the efficacy of certain therapies, such as platelet-rich plasma, remains controversial, necessitating further clarification of their optimal indications.

In donor site management, cell suspensions and micrografting techniques represent an advanced direction for minimizing patient trauma by significantly reducing the donor site area, but it should be noted that they may not be superior to standard care in specific chronic wounds like well-vascularized diabetic foot ulcers (44); moist healing dressings remain the foundational choice for accelerating donor site healing. Minimally invasive techniques like ultra-thin skin grafting also help reduce donor site morbidity and pain (46). Topical agents like pirfenidone (47), birch bark extract (48), and Alkannin ointment (49) show promise in accelerating donor site healing.

For chronic, hard-to-heal wounds, acellular fish skin grafts demonstrate superior healing outcomes and cost-effectiveness compared to standard care.

In scar management, clinical decisions necessitate a clear trade-off between short-term efficacy (e.g., pain control, healing speed) and long-term aesthetics. The application of technologies such as silver-containing dressings and ADMs should be based on this trade-off and discussed with the patient. Birch bark extract gel, which improves long-term skin appearance while promoting healing (48), represents a potential option for scar management. Allogeneic skin and Moist Exposed Burn Ointment have shown advantages in improving scar quality.

### 7.3 Adjunctive therapies and technological innovations continuously expand treatment boundaries

Physical adjunctive therapies offer effective complements for specific complex scenarios. Hyperbaric oxygen therapy enhances graft survival and accelerates donor site healing; non-invasive energy-based therapies like extracorporeal shock wave, low-level laser, and non-contact low-frequency ultrasound can accelerate epithelialization and alleviate symptoms; NPWT has clear advantages in complex wound management, but its value in relatively straightforward wounds lies more in functional rehabilitation. When selecting an NPWT system, cost-effectiveness analysis should inform the decision if efficacy is comparable (54). In functionally critical areas, functional rehabilitation should be prioritized, and the value of NPWT may transcend its healing effects; tissue expansion techniques can effectively avoid donor site morbidity. Innovations in skin harvesting (e.g., molecular resonance generator) and dressings facilitating early mobilization (e.g., Spincare®) contribute to early functional recovery.

Systemic perioperative management is fundamental to ensuring skin graft success. Preoperative prophylactic strategies like tissue expansion can minimize the need for grafting; intraoperative technical optimization directly impacts surgical efficiency and early prognosis; postoperative systemic interventions, such as targeted antibiotic use and patient education, are crucial for graft survival. Patient adherence is also a key factor in long-term wound management (45); novel hemostatic agents require balancing efficacy with cost (33); liposomal bupivacaine did not demonstrate additional analgesic benefits over lidocaine (34); propranolol may reduce bleeding and accelerate donor site healing (35).

### 7.4 Research gaps and future prospects

Current research has several gaps: the mechanism behind the inconsistent efficacy of platelet-rich plasma across different wound types remains unclear; the lack of demonstrated superiority for cell therapy in chronic wounds (such as diabetic foot ulcers) (44) suggests its applicable scenarios still need precise definition; most studies have short follow-up periods, with insufficient evaluation of long-term function and scar quality. In the future, with advancements in tissue engineering, regenerative medicine, and intelligent biomaterials, postoperative management of skin grafts is expected to move towards a more personalized and precise phase, ultimately achieving the ideal integration of wound healing with functional and aesthetic restoration.

To achieve true precision and individualization in nursing care, future research should focus on the following key areas: Firstly, strengthening patient-centered long-term outcome assessments, particularly long-term follow-up of scar quality, functional recovery, and quality of life. Secondly, conducting more in-depth health economic analyses to inform resource allocation. Thirdly, optimizing patient selection and treatment protocols, defining the optimal indications for various advanced technologies, and developing and validating minimally invasive grafting techniques that can be safely implemented in outpatient or community settings. Finally, combining molecular biology approaches to identify biomarkers of healing and scar formation, guiding the development of optimal treatment strategies.
